# Adipocytes in Breast Cancer, the Thick and the Thin

**DOI:** 10.3390/cells9030560

**Published:** 2020-02-27

**Authors:** Ilona Rybinska, Roberto Agresti, Anna Trapani, Elda Tagliabue, Tiziana Triulzi

**Affiliations:** 1 Molecular Targeting Unit, Department of Research, Fondazione IRCCS Istituto Nazionale dei Tumori, Milan 20133, Italy; ilona.rybinska@istitutotumori.mi.it (I.R.); elda.tagliabue@istitutotumori.mi.it (E.T.); 2 Division of Surgical Oncology, Breast Unit, Fondazione IRCCS Istituto Nazionale dei Tumori, Milan 20133, Italy; roberto.agresti@istitutotumori.mi.it (R.A.); anna.trapani@istitutotumori.mi.it (A.T.)

**Keywords:** adipocytes, breast cancer, obesity, adipokines, cancer associated adipocytes (CAAs), cachexia

## Abstract

It is well established that breast cancer development and progression depend not only on tumor-cell intrinsic factors but also on its microenvironment and on the host characteristics. There is growing evidence that adipocytes play a role in breast cancer progression. This is supported by: (i) epidemiological studies reporting the association of obesity with a higher cancer risk and poor prognosis, (ii) recent studies demonstrating the existence of a cross-talk between breast cancer cells and adipocytes locally in the breast that leads to acquisition of an aggressive tumor phenotype, and (iii) evidence showing that cancer cachexia applies also to fat tissue and shares similarities with stromal-carcinoma metabolic synergy. This review summarizes the current knowledge on the epidemiological link between obesity and breast cancer and outlines the results of the tumor-adipocyte crosstalk. We also focus on systemic changes in body fat in patients with cachexia developed in the course of cancer. Moreover, we discuss and compare adipocyte alterations in the three pathological conditions and the mechanisms through which breast cancer progression is induced.

## 1. Introduction

In cancer, the tumor surrounding cells have altered biological properties compared with the normal state. We know today that there is a large spectrum of reciprocal interactions between a tumor and its stroma that significantly influence tumor biology and support tumor progression. Given the close juxtaposition of adipocytes and breast cancer (BC) cells, it is somehow surprising that knowledge-intensive research about the role of adipocytes in tumor initiation, growth, and metastasis is a relatively new area of investigation [1]. The previous lack of attention can be partially explained by the fact that adipose tissue (AT) used to be regarded as a rather inert site principally storing energy in the form of lipids and cushioning the body [2]. It has taken on a new significance, particularly since the discovery of leptin in 1994 [3]. Thereafter, the list of adipocyte-derived factors has been increasing at an extraordinary pace, and the traditional role of AT has progressed to a fully functioning endocrine organ, acting on local and systemic levels and modulating feeding behavior, total body energy expenditure, andsuch fundamental processes as hematopoiesis, lymphopoiesis, immune function, and reproduction [4,5]. A great importance attributed to the role of adipose tissue in cancer is sustained by two main observations: (i) epidemiologic studies have demonstrated an association between obesity and some cancers [4], and (ii) many tumors metastasize to adipose rich niches such as abdomen or bone marrow [6,7]. In addition, adipose mass in these sites is increased with obesity and aging and in turn may explain cancer progression in obese and/or elderly patients [7]. Worth mentioning, cancer cachexia, which is due to severe metabolic dysregulation in fat tissue, is observed in more than 50% of cancer patients, including those with BC [8]. Importantly, adipose tissue wasting observed in cancer cachexia shares some similarities with stromal-carcinoma metabolic synergy [9]. Finally, it is obvious that, in organs such as the breast, early local tumor invasion results in immediate cancer cell proximity to adipocytes. The large number of recently published studies supported the presence of the heterotypic interaction between epithelial cells and adipocytes at the invasive tumor front and, together with different variants of the two-compartmental in vitro models, provided more details about the mechanisms of this vicious interaction. The aim of this article is to summarize current knowledge on the role of AT and adipocytes in BC progression, focusing on mechanisms and implications of obesity, closeness between tumor cells and adipocytes, and cachexia.

## 2. Adipogenesis and the Adipose Organ

### 2.1. The Adipogenesis Process

Adipocytes derive from multipotent mesenchymal stem cells that also have the potential to differentiate in myoblasts, osteoblasts, and chondroblasts [10]. Molecular characteristics, based mainly on in vitro studies, divide adipogenesis into two stages. The first step, named determination, defines cell fate. This stage requires the commitment of a pluripotent stem cell to the adipocyte lineage. Entrance into this stage of differentiation was once considered non-reversible, but now we know that this process is multidirectional, and adipocytes under certain stimuli can return to a phenotypically stem-like precursor state. Cell growth arrest initiates the second phase of adipocyte differentiation, during which preadipocytes begin to resemble characteristics of the mature fat cells in terms of expanded lipid metabolism and the secretion of adipocyte-specific proteins. Adipogenesis in vitro is based on a cocktail of inducing agents containing 3-isobutyl-1-methylxantine (IBMX) (stimulating the cAMP-dependent protein kinase pathway), dexamethasone (stimulating the glucocorticoid receptor pathway), and insulin (serving as permissive differentiation signal to preadipocytes) required to induce differentiation of preadipocytes into fat cells [10]. Adipogenesis in its complexity is a highly regulated process, at the head of which stand the nuclear receptor-peroxisome proliferator-activated receptor-γ (PPARγ) as well as transcription co-activators CCAAT/enhancer-binding protein α and β (C/EBP α and C/EBP β) [11]. Enhanced lipid accumulation and subsequent expression of the adipocyte fatty-acid binding protein (*FABP4*) and the insulin-responsive glucose transporter type 4 (*GLUT4*) are characteristic of an early stage of differentiation [10]. Mature adipocytes additionally express adiponectin (*ADIPOQ*), leptin (*LEP*), the adipose triglyceride lipase (*ATGL*) and lipoprotein lipase (*LPL*), as well as high levels of perilipin 1 (*PLIN1*), a lipid droplet coating protein.

### 2.2. The Adipose Tissue

Classification, based mainly on cellular mitochondrial content, divides adipocytes in white, beige, and brown types. White adipocytes are dominant in the body and contain a large, unilocular lipid droplet and are specialized for storage of neutral lipids. Due to their unique expression of a mitochondrial membrane protein called uncoupling protein 1 (UCP1), brown adipocytes can generate endogenous heat in a process called thermogenesis. Classification of white/brown adipocytes does not fully describe their diversity as, even among white adipocytes, cells from different locations have distinct molecular and physiological properties.

Based on the anatomical distribution, AT may be divided into subcutaneous, visceral, intramuscular, bone marrow, and dermis subtypes [12]. There are significant gender-related differences in the expansion of the AT mass, but, in general, human subcutaneous AT comprises ~80% of total body fat and is located primarily in the gluteal and the femoral depots. Additionally, in women, the breast fat pad is a relevant contributor to total subcutaneous fat. On the other hand, the visceral fat portion includes omental, mesenteric, and epiploic adipose tissue as well as gonadal, epicardial, and retroperitoneal fat pads. It surrounds vital organs and makes up approximately 5% to 20% of total body fat in normal weight individuals. Although adipocytes are the major component of AT, it is composed also by preadipocytes and a stromal-vascular fraction containing endothelial cells (10–20%), pericytes (3–5%), stem and progenitor cells (0.1%), as well as a rich collection of innate and adaptive immune cells [macrophages, dendritic cells, mast cells, eosinophils, neutrophils, and lymphocytes (5–45%)] [4]. Curiously, every fat depot is really unique. More to the point, differences concern preadipocyte populations, signals governing adipogenesis, metabolic functions (secretion of adipokines and inflammatory cytokines), lipolysis rates and thermogenic potential, degree of innervation, and vascularization [10]. This is in line with their differential involvement in metabolic-related complications. Worthy of consideration, visceral fat is strongly associated with metabolic disease risk, whereas subcutaneous adiposity is comparatively benign [13].

### 2.3. Mammary Adipocytes

Physiological intimate bidirectional interaction of mammary adipocytes with adjacent epithelium leading to an extreme plastic phenomenon occurring in adult tissue is a hallmark of mammary adipocytes. Indeed, fat cells are required for proper mammary duct development during puberty as well as for the maintenance of ductal architecture in the adult mammary gland [14]. Moreover, during pregnancy, they modify their phenotype, making a place for alveolar cells able to synthesize various milk constituents [15]. The fate of adipocytes during this process remains hotly discussed. In particular, whether fat cells die off or transdifferentiate into another cell type, as suggested by Morroni et al. [16] is unknown. Maybe they remain constantly present as smaller, slimmed cells, giving the impression of adipocytes disappearance [16,17]. Considering the above, the post-lactation adipogenesis seems to be a process not involving proliferative events but rather based on lipid trafficking and cytoplasm refilling with components derived from regressing milk-producing cells [18]. Nowadays, we know such particular dialog persists also in pathological conditions, such as cancer.

Tumor cells exert significant effects on adjacent adipocytes. The histological picture of tumor-surrounding adipocytes unraveled that they become fewer, lose lipids, and acquire fibroblast-like features [19]. Several in vitro studies using two compartmental models (cancer cells/adipocytes) supported that the phenotype of adipocytes observed at the invasive tumor front was the result of direct influence of tumor cells [20]. The intimate cross-talk between cancer cells and adipocytes resulted in the initiation of the process of adipocytes dedifferentiation in the sense of a reduction of adipocytes terminal differentiation with a reduction in the expression of differentiation markers such as PPARγ and C/EBPα as well as their downstream genes such as *FABP4*, *ADIPOQ*, and hormone sensitive lipase (*HSL*) [19,21]. Tumor modified adipocytes (named cancer associated adipocytes, CAAs, Table 1) may be an intermediate form of arising cancer-associated fibroblasts (CAFs), which constitute the non-homogenous population comprising the majority of stromal cells in BCs [22]. Adipocyte-derived fibroblasts exhibited increased expression of the CAF marker fibroblast-specific protein-1 (FSP-1) but not alpha smooth muscle actin (a-SMA) [22].

Although some candidate molecules secreted by tumor cells such as tumor necrosis factor alfa (TNF-α) [36], Wnt3a [22], Wnt5a [37] and stromelysin-3 (MMP11) [38] have been proposed to dedifferentiate mature adipocytes, the precise mechanisms that could be involved in tumor-driven adipocyte dedifferentiation and lipid loss remain to be discovered.

## 3. Epidemiological/Clinical Association between Obesity and BC

According to the World Health Organization (WHO) and the National Institute of Health (NIH), overweight and obesity are clinically present when the body mass index [BMI, defined as weight (kg)/ height (m^2^)] is greater than 25 or 30 kg/m^2^, respectively [39]. Almost two billion adults and more than 500 million people are respectively defined as overweight and obese in the world, and these rates will increase in the future [40,41].

BC is the most frequent female type of cancer and a leading cause of cancer-related mortality worldwide [42], and it is a highly heterogeneous disease with a wide range of hysto-pathological, biomolecular patterns, and clinical behaviors that associate with different prognosis [43]. Leaving aside genetic predispositions, such as BRCA 1–2 mutations, or reproductive factors, as BC causes, tumor pathogenesis is a multifactorial process in which metabolic consequences and related interactions of an unhealthy lifestyle are epidemiologically and clinically widely studied. Surely, it is considered interesting and challenging that unbalanced diet, unsatisfactory physical activity, and high alcohol consumption contributing to determine a high BMI may be modifiable risk factors, as shown in the European Prospective Investigation into Cancer and Nutrition (EPIC) Italy study on over 15,000 post-menopausal women [44].

Two of the leading questions in this area of investigation are if there is a linear relation between increasing BMI and BC onset and what subtypes of BC are more influenced by obesity. Epidemiologically, obesity is a risk factor for many cancers [45], and it is particularly associated with BC in post-menopausal women. In a prospective cohort study within the Nurses’ Health Study, more than 87,000 women were followed up, recording their weight change during a long-observed period of life and showing that weight gain since menopause significantly increases the risk of BC, particularly in obese women [46]. Other convincing evidence that body fatness and weight gain may be directly and progressively related to post-menopausal BC has been described in the larger European EPIC study on almost 250,000 post-menopausal women in which, conversely, healthy behaviors reduced the risk of BC [47]. Furthermore, evaluating in a meta-analysis the relationship of adult weight gain with subgroups of BC, Vrieling at al. showed in obese patients a significantly increased risk of post-menopausal estrogen receptor (ER)+BC [summarized risk estimate (RE) = 2.33; 95% confidential interval (CI) 2.05–2.60] [48]. This association between BMI and ER+ BC was also demonstrated by an analysis of pooled tumor markers and epidemiological risk factors in more than 35,000 invasive BC patients from 34 studies participating in the Breast Cancer Association Consortium [49].

In pre-menopausal women, studies examining the association between diet, BMI, and BC showed inconsistent results with major complexity. Suzuky et al. associated a high BMI with a 20% lower risk for ER+ BC in pre-menopausal women (95% CI = −30% to −8%), confirming an 82% higher risk in post-menopausal women (95% CI = 55–114%) [50]. The same authors showed that each five unit increase in BMI was associated with a 33% increased risk among post-menopausal women (95% CI = 20–48%) and a 10% decreased risk in pre-menopausal women (95% CI = −18% to −1%). In this paper, no association was observed for ER-negative BC, but further meta-analysis showed a significantly increased risk of triple-negative BC (TNBC) in pre-menopausal women with high BMI [51].

Some meta-analyses analyzing large numbers of patients showed that obesity is also positively related to BC recurrence and mortality. Protani et al., evaluating 43 historical studies (which principally defined obesity using BMI more than waist-to-hip ratio) and comparing obese versus non-obese patients, showed a statistically significant higher risk for overall survival (HR = 1.33) and for BC specific survival (HR = 1.33; 95% CI = 1.21–1.47) in obese patients [52]. In interest, differences were seen in pre-menopausal (HR = 1.47) and in post-menopausal (HR = 1.22), even if they were not statistically significant. A more recent meta-analysis on 82 studies evaluating more than 210,000 patients showed that BMI was significantly associated with BC mortality; for BMI calculated before the diagnosis, the summary relative risks of total mortality and of BC mortality were 1.41 (95% CI = 1.29–1.43) and 1.35 (95% CI = 1.24–1.47) for obese compared with normal weight patients, with a difference for pre-menopausal (1.75; 95% CI = 1.26–2.41) and for post-menopausal (1.34; 95% CI = 1.18–1.53) BC patients [53]. Conversely, in a further systematic review and meta-analysis, weight gain (≥ 5% of body weight, measured at least one year post-primary BC diagnosis) versus maintaining body weight during the follow-up in BC patients seemed to be associated with higher all-cause mortality but not with hazard of BC specific mortality [54]. Furthermore, in a study on 15,000 patients, overweight and obesity have been significantly associated with higher probability to develop controlateral BC (adjusted hazard ratio: 1.50; 95% CI = 1.21–1.86) compared to normal weight women after 10 years of follow-up [55].

Obviously, obese patients may have delayed diagnosis with more advanced disease at diagnosis compared with patients with normal BMI. This was shown by a study in Denmark on over 18,000 patients in which obese patients were older and had significantly more advanced BC at diagnosis than normal weight patients. However, after data adjustment for disease characteristics, obesity remained an independent prognostic factor for distant metastases and for death in BC [56]. Similarly, a French study on over than 14,000 BC patients showed that, even if obese patients presented more advanced tumors at diagnosis, multivariate analysis identified a relevant independent effect for obesity on BC recurrence [57].

## 4. Evidence of the Pro-Tumorigenic Effect of the Adipocyte-Tumor Cell Crosstalk

Several in vitro studies have demonstrated that AT supports and promotes tumor growth. Moreover, the degree of tumor infiltration into the adjacent fat AT serves as histological criterion reflecting the aggressiveness of the tumor and is indicative of poor prognosis [58,59]. A special role in this matter can be attributed to mature AT fraction. It was evidenced that mature adipocytes being in direct contact with BC cells, but not stromal AT preadipocytes, increased tumor growth of ER+ BC cell lines. Importantly, it is likely that mature adipocytes, during coculture with BCs for a relatively long time (7 days), acquire tumor promoting characteristics and become CAAs. This would be in line with a study carried out by Dirat et al. [19], which clearly showed that cross-talk between the two cell populations is necessary to observe the pro-invasive effect. In detail, only CAAs previously conditioned by tumor cells, not “naive” adipocytes, release molecules stimulating tumor invasiveness. Although a growth-promoting effect has been largely reported in several models, e.g., ovarian, prostate, colon, and melanoma cancers [21], the interaction between adipocytes and BC cells is very complex and often ambiguous (Table 2). For example, some BC cell lines cocultured with adipocytes exhibit increased proliferation but others did not. It can suggest that the effect depends on the tumor subtype. In contrast to proliferation, the adipocyte effect on cancer migration/invasion seems to be clearer and more repeatable in various studies. This in line with experimental studies that have shown that cocultures of adipocytes with BC cells increased cancer cell migration [60] and invasion [19]. Of note, the list of studies clearly shows that the aggressive-promoting effect of adipocytes on cancer cells is not conditioned by the direct contact, suggesting that it is caused by soluble molecules. The cancer-adipocyte crosstalk is of great importance in the modulation of tumor behavior and induction of partial epithelial mesenchymal transition (EMT) [60,61]. It was also shown that some BC cell subtypes, when cocultured with mature adipocytes, displayed a downregulation of the epithelial marker E-cadherin with a simultaneous morphological change (spindle shape) [60]. In contrast, preadipocytes were shown to induce expression of E-cadherin in BCs cell lines. In the same study, however, adipocytes did not affect E-cadherin expression in cocultured BC cells.

Numerous studies demonstrated that adipocyte lipolysis stimulated by cancer cells is at the very heart of the synergy between cancer cells and adipocytes. Lipolysis is defined as the hydrolytic cleavage of ester bonds in triglycerides (TGs), resulting in the generation of fatty acids (FAs) and glycerol [63]. BC cells, when cocultured with adipocytes, accumulate lipids [21]. Using the adipocyte-ovarian cancer cell coculture model, the direct transfer of lipids from adipocytes to ovarian cancer cells was proved. Moreover, it was shown, perhaps by induction of FA β-oxidation (FAO) in cancer cells, to promote tumor growth, suggesting that adipocytes act also as an energy source for cancer cells [21].

## 5. Mechanisms Behind the Pro-Tumorigenic Effect of Adipocytes

The pathological expansion of altered white AT in obesity leads to an abundant production of several biologically active factors, including inflammatory cytokines, hormones, adipokines, and lipid metabolites that can be considered mediators between obesity and cancer. Recent evidence demonstrated that CAAs have an altered secretome compared to mature adipocytes. CAAs share some common features with obese adipocytes, e.g., they secrete significantly higher levels of motility factors such as CCL2, CCL5, autotoxin (ATX), as well as proinflammatory cytokines such as IL-1β, IL-6, TNF-α, VEGF, and leptin. Moreover, cachectic adipocytes give an essential contribution to cachexia (Table 3).

### 5.1. Inflammation

It is well recognized that chronic inflammation with high circulating levels of C-reactive protein represents a pathophysiological condition that bridges obesity and cancer [95]. However, local inflammation driven by altered adipocyte also has a role in obesity. The histological biomarker of this local inflammation is represented by the presence of the so-called “crown-like structures” (CLSs), derived from dead/dying adipocytes surrounded by macrophages. Macrophages are recruited in the obesity-associated AT due to excessive accumulation of fat and production of chemokines such as CCL2 and CCL5 [70,73] and are switched towards an M1 proinflammatory state [96]. A paracrine loop involving free fatty acids (FFAs) and tumor necrosis factor-alpha (TNF-α) between adipocytes and macrophages establishes a vicious cycle that leads to high secretion of cocktail of proinflammatory mediators such as prostaglandin E2 (PGE2), TNF-α [64], IL-1β [69], and IL-6 exacerbating inflammatory status in the AT. This phenomenon has been described mainly in the visceral fat of obese women, but it occurs also in the breast AT and has a role in BC progression. Indeed, CLSs are enriched in BCs of obese patients and have negative impact on disease recurrence and survival [97]. Moreover, inflammatory factors (e.g., IL-6, IL-1β, TNF-α) are found at high levels in obese patients and are associated with poor outcome in BC patients [98]. The presence of CLSs is not limited to obese patients, but it also occurs in BCs from normal weight women [99]. Tumor trace released on adipocytes provides important modifications of fat cell secretome, which, generally speaking, becomes highly inflammatory. Indeed, CAAs secrete significant amounts of pro-inflammatory mediators such as CCL2 and IL-1β, leading to the accumulation of macrophages forming CLSs. Notably, in normal weight BCs, CLS are associated with reduced survival, supporting a role for local AT inflammation in BC progression [100,101].

#### IL-6 and TNF-α

In normal AT, adipocytes are not the major source of IL-6, however, under pathological conditions such as obesity and cancer, the levels of IL-6 secreted from adipocytes increase significantly [67,68]. Changes in adipocyte secretory profile (e.g., TNF-α, IL-6, and IL-1β) were also observed after coculture with BC cells or in isolated adipocytes from BCs [20]. Activated adipocytes assume an inflammatory phenotype (CAAs) and release more IL-6 [1,19]. Function of IL-6 in breast and other solid tumors progression and treatment was shown to be associated with the development of stem cell phenotype, angiogenesis, cachexia, and resistance to therapy [102]. One-third of the total circulating IL-6 is expressed predominantly by adipocytes. IL-6 circulating levels increased in human obese subjects and correlated with adiposity [67,68]. In BC patients, the extent of the increase of IL-6 in serum was correlated with poor disease outcome and reduced prognosis [103].

TNF-α is an important inflammatory factor in the tumor microenvironment that is generated by tumor and stromal cells. Moreover, BC cells in coculture with adipocytes stimulated their TNF-α production [19]. While, in the serum of healthy women, TNF-α is generally not detected, clinical studies have reported high levels of this cytokine in patients with BC [104]. It was evidenced that TNF-α may play an important role in BC development and proliferation, chemoresistance, angiogenesis, invasion, and metastasis [105]. Through upregulation of IL-6 and aromatase expression, TNF-α participates with the comprehensive regulation of estrogen synthesis [106]. In addition, TNF-α as a lipolytic agent was shown by activation MEK, ERK, and elevation of intracellular cAMP to enhance lipolysis in human adipocytes [107].

### 5.2. Estrogens

More than 75% of BCs express the ER, which means the vast majority of BCs grow and progress in response to estrogens. Of note, estradiol (E2), through the alteration of the tumor microenvironment, was shown to increase the growth of ER-negative BCs [108]. Estrogen biosynthesis is catalyzed by the enzyme aromatase. Most of the estrogen in pre-menopausal women is synthesized by the ovaries, while, after menopause, the adipose organ becomes the predominant source of aromatase expression and estrogen production [109]. Given the close proximity of fat tissue to epithelial cells in mammary gland tissue, aromatase derived from breast adipose stromal cells may have a substantially higher impact on breast carcinogenesis than aromatase expressed in other parts of the body. Indeed, estrogen levels in BCs are as much as 10 times greater than in the circulation of post-menopausal women [110]. It is therefore likely that locally produced estrogens, as a consequence of breast AT inflammation, may represent a key driver of post-menopausal BCs in obesity. Accordingly, aromatase activity is higher in AT of obese than normal weight women [75]. Even if direct evidence is still lacking, considering that aromatase expression in AT is a marker of preadipocytes rather than mature adipocytes and that stimulation of PPARγ leads to a reduction in aromatase expression [111], it is likely that dedifferentiated CAAs are relevant sources of aromatase within the tumor microenvironment. In support, aromatase activity was found higher in the breast quadrant containing the tumor than in the opposite one [76]. It is interesting that factors inhibiting adipocyte differentiation, such as IL-6 and TNF-α, at the same time stimulate aromatase expression in AT.

### 5.3. Adipokines

#### 5.3.1. Adiponectin and Leptin

Adiponectin is the most abundant protein secreted by AT with wide range influence on several tissues and organs, performing insulin-sensitizing, anti-inflammatory, antiatherogenic, proapoptotic, and antiproliferative actions [112]. Unlike most of the other AT-derived proteins, serum adiponectin is reduced in obesity [80]. It is reduced also in CAAs in vitro and at the invasive front compared with normal mammary AT [19,30]. Adiponectin released by adipocytes in the BC microenvironment has a protective effect against tumorigenesis mainly through intracellular mechanisms initiated via its receptors (AdipoR1 and AdipoR2). It has been extensively described that adiponectin attenuates growth and invasion of BC cells and induces apoptosis by activation of AMPK and PI3K/AKT and ERK1/2 inhibition [113] rather than inducing autophagic cell death [114]. While consistent results on its anti-tumor activity have been obtained in ER-negative BC cells, controversial have been reported in ER+ BC cells [115].

Leptin is a hormone made by AT physiologically acting primarily on neurons in the hypothalamus, regulating food intake and energy expenditure. The role of leptin in tumorigenesis was suggested by the high expression of its receptor (ObR) in several cancer cells. Both leptin and ObR were found overexpressed in BCs, especially in higher grade tumors, and were shown to be related with distant metastasis and poor prognosis [116]. Prevalently, leptin exerts its biological function through binding to its receptor, which activates multiple downstream signaling pathways such as STAT3, ERK, and PI3K signaling, involved in the control of cell proliferation, differentiation, survival, migration, and invasion [117]. Leptin signaling was also shown to be involved in the promotion of a stem-like phenotype related to resistance to therapy and tumor recurrence and metastasis [118]. Its pro-tumorigenic activity in BCs also derived from its interaction with the ER signaling. Indeed, leptin works on two levels; on one hand, it modulates estrogen production in adipose stroma, and on the other hand, it induces upregulation of ER expression and ER functional transactivation in BC cells governing estrogen sensitivity in cancer cells [119]. Accordingly, it has been shown that obese stromal cells producing large quantities of leptin increased proliferation and metastasis of several ER+BC cell lines through a leptin-mediated pathway [120]. Leptin was described to have a pro-tumorigenic role modulating the tumor immune microenvironment. Indeed, it was shown to induce STAT3 activation in tumor infiltrating effector T cells promoting FAO, which in turn facilitates the use of FAs as an energy source, impedes glycolysis associated with restricted CD8+ T cell antitumor function, and enhances BC progression [121]. Moreover, leptin has been described to mediate dysfunction of CD8+ T cells by increasing the expression of PD-1 [122]. These effects have clear implications in the efficacy of checkpoint inhibitors in obese patients.

Leptin is a key paracrine mediator regulating the interaction between stromal cells and BC cells in the meaning of tumor metabolism. It was shown that leptin released by AT contributes to the metabolic features associated with BC malignancy, such as switching the cells’ energy balance from mitochondrial β-oxidation to the aerobic glycolytic pathway [123]. Obesity impacts leptin [77,78] and adiponectin levels in opposite manners, which means that not their absolute quantities but rather the mutual proportion in which they occur may be a key parameter indicating the relative risk of BC. Indeed, high leptin-to-adiponectin ratio is associated with increased risk of post-menopausal BC [124] and with increased progression in TNBC [125]. In addition, its expression characterized CAAs of BC tissue [20] and mammary fat tissue from BCs compared to fat derived from benign lesions [30].

#### 5.3.2. Autotaxin and Resistin

Autotaxin (ATX) is a secreted glycoprotein produced by platelets, endothelial cells, fibroblast, adipocytes and, in varying degrees, by cancer cells [20]. Enzymatic activity of ATX converts lysophosphatidylcholine (LPC) into lipid signaling molecule lysophosphatidic acid (LPA), which controls key processes such as cell renewal, cell migration, proliferation, and survival [126]. Increased ATX expression in tumors upregulates inflammatory programs and is associated with enhanced tumor progression, aggressiveness, increased angiogenesis, metastasis, and chemoresistance [127]. Benesch et al. [85] proposed a special role for the adipose compartment within the BC microenvironment in the supply of ATX. Namely, inflammatory signals secreted by tumor cells increase ATX expression in CAAs and fibroblasts, reinforcing the inflammatory vicious cycle and contributing to tumor progression. The pro-tumorigenic crosstalk between the tumor and the surrounding AT was shown to be interrupted by ATX inhibition. ATX is highly expressed in subcutaneous fat depots of obese patients [82] and is increased in circulation of patients with higher BMI [83,84].

Resistin is a fat-derived secretory factor usually found in inflammatory zones [128]. Recently published data evidenced that resistin promotes the metastatic potential of BC cells by inducing EMT and stemness [129]. As with other adipokines circulating in the blood, resistin may act on BC systemically and locally. It was found to be higher in the circulation of obese patients and ob/ob mice, a well-described model of obesity [67]. Also, CAAs in BC tissues produce this metastasis-inducing adipokine [20]. The resistin receptor adenylyl cyclase-associated protein 1 (CAP1) was shown to be expressed by numerous BC cell lines and primary human tumors. Moreover, its high expression in BC patients was associated with characteristics of aggressiveness and poor prognosis [130].

#### 5.3.3. HGF and IGF

Adipocytes and preadipocytes are known to secret hepatocyte growth factor (HGF), which, together with its receptor present on tumor cells (c-Met), forms a signaling pathway intensely correlated with proliferation, metastasis, and angiogenesis [131]. HGF was shown to be highly secreted by adipocytes derived from obese individuals; accordingly, its serum level was higher in obese subjects when compared to lean subjects [86]. Of note, it was shown that c-Met expression was increased at the BC invasive front in adipocytes proximity. Although cancer cells seem to not increase expression of HGF in nearby adipocytes, the fact that expression of HGF and c-Met at the interface of adipocytes and cancer cells was correlated with histologic grade and reduced patient survival is certainly an important indication that the pathway initiated by the adipocytes-derived HGF has an impact on tumor progression [132].

Insulin-like growth factor 1 (IGF-1) is also secreted by adipocytes and preadipocytes, and its release is increased about two-fold in both undifferentiated cells and differentiated adipocytes from obese compared to lean individuals [87,88]. IGF-1, through the binding of its receptor IGF-1R expressed in tumor cells, activates PI3K/AKT and MAPK pathways, resulting in their enhanced proliferation. Suppression of IGF-1R in BC cells was shown to inhibit the tumor promoting effect of adipocytes.

### 5.4. Extracellular Matrix Remodeling

Cancer progression builds upon the ability of cancer cells to traverse the extracellular matrix (ECM) barrier access the circulation and establish distant metastases. The ECM composition not only orchestrates cancer and stromal cell behavior but also regulates the spectrum of reciprocal complex interactions between cells. It is widely recognized that both biochemical and biomechanical properties of the ECM influence cancer cell plasticity, allowing them to survive in hostile microenvironments and resist therapy. Production of ECM components functioning as signaling molecules and its continuous remodeling are clearly implicated in tumor progression. In AT, ECM is crucial for maintaining the structural integrity of adipocytes and plays a pivotal role in adipogenesis. Adipocytes are an abundant source of ECM components. However, tumor-induced adipocyte “activation” makes them produce even larger quantities of tumor promoting ECM components, such as collagen VI (COLVI) [19,21], which was described to contribute to AT fibrosis and inflammation in obesity [91,94]. COLVI was shown to promote tumor growth and survival signaling through the NG2/chondroitin sulfate proteoglycan receptor expressed on tumor cells [133]. The cleaved fragment of the COLVI α3 chain—endotrophin—acts a potent profibrotic factor stimulating TGF-β-dependent EMT [134].

In the same way, adipocytes at the invasive front of human tumors and CAAs in vitro exhibit increased expression of MMP11) [19,38]. MMP11 is a potent physiologic negative regulator of adipogenesis, and its expression in adipocytes was shown to be induced by tumor cells, in turn leading to the accumulation of non-malignant peritumoral fibroblast-like cells, promoting cancer cell survival and tumor progression. Thus, MMP11 plays the central role during tumor desmoplasia [135].

### 5.5. Metabolic Shift

Cancer cells invading regions of adipocytes in the tumor microenvironment interfere with essential functions of white AT, such as controlling energy balance, leading to the redistribution of nutrients in favor of cancer cells. It was shown that a tumor educates its stroma by downregulating p62, and such a prepared stroma shuts down energy utilization, which increases nutrient availability for cancer cells [136]. Recently, much attention has been paid to the role of adipocytes releasing FAs and other macronutrients supporting tumor growth [9,137]. Tumor cells switching from glycolysis to lipid-dependent energy production are supported by both de novo lipogenic synthesis and the acquisition of exogenous FAs [138]. FAs provide an excellent source of energy production through the FAO. Cancer cells can also store excess lipids in the form of lipid droplets, which supply energy to power their expansion and metastasis [138]. Especially, obese adipocytes supply more FAs to cancer cells than non-obese adipocytes, increasing the energy amount available for tumor growth and metastasis [34]. Cell surface fatty acid translocase (CD36) has been recognized as a marker of metastasis initiating cells in various cancer types, including BC [138,139]. Those cells are highly responsive to exogenous FAs, and the inhibition of CD36 was shown to impair metastasis [138,140]. FFAs released from CAAs serve not only as nutrients but can also be used for the biosynthesis of a series of lipid-signaling molecules that promote tumorigenesis [9].

Tumor-educated adipocytes due to strong metabolic pressure shift their metabolism to glycolysis with concomitant release of energy-rich metabolites, such as lactate and pyruvate. The dynamic monocarboxylate shuttle between BC cells and adipocytes through monocarboxylate transporters (MCT) plays a role in BC aggressiveness [141]. Indeed, it was shown that adipocytes co-cultivated with tumor cells exhibited upregulated expression of 4MCT4, facilitating lactic acid efflux [142]. Cancer cells, on the other hand, are equipped with an appropriate apparatus for lactic acid uptake, such as MCT1 and MCT2. In fact, highly proliferating ER-negative BC subtypes express high levels of MCT1, which is associated with poor patient outcome [141]. The association was even stronger for combined expressions of MCT4 and MCT1 in AT and tumor cells, respectively.

Ketone bodies produced and released by glycolytic adipocytes are an ideal substrate for ATP production by driving oxidative mitochondrial metabolism in invasive cancer. They may burn more efficiently than other mitochondrial fuels, even during hypoxia, potentially allowing the tumor to grow in the absence of an optimal blood supply [143]. Importantly, the coexistence of adipocytes and tumor cells potentiates both ketogenesis in adipocytes as well as ketolytic activity in BC cells [142]. Additionally, it was shown that β-hydroxybutyrate secreted from adipocytes enhanced BC cells malignancy in vitro, upregulating several tumor-promoting genes in BC cells [144]. Induction of ketone-specific gene signature was shown to be associated with worse outcomes in BC patients [145].

## 6. Adipocytes in Cancer-Associated Cachexia

Cancer-associated cachexia is a well-known complication of cancer that frequently is the cause of death in cancer patients. As a systemic illness, cachexia affects the vast majority of patients with end stage cancer. Due to an energy imbalance condition and inflammation, the body burns its own components to sustain the tumor growth. Cachexia differs from malnutrition in as much as cachexia cannot be successfully treated by supplemental nutrition alone [145]. Cachexia has been most frequently recognized in the course of pancreatic, gastric, colorectal, lung, head, and neck cancers [146]. Although in the past cachexia was not often documented in BC patients, more recent studies suggest its particular impact on bone metastasis common in patients with advanced BC [147]. The previously rather neglected AT dysfunction is an essential contributor to cachexia and has been recognized as an early phenomenon occurring just before the skeletal muscle atrophy [148,149]. This is in line with observations carried out in tumor-bearing mice, which confirmed that AT wasting is an early event occurring at a time when the tumor is hardly palpable [150]. Moreover, the breakdown of adipocyte triacylglycerols (which is at the basis of cachexia-associated fat tissue remodeling) may actually activate muscle proteolysis [151]. Several serum factors such as TNF-α, IL-1β, IL-6, and a zinc-glycoprotein (ZAG), also called lipid-mobilizing factors secreted by tumor or host cells, have been shown to be involved in local as well as systemic AT lipolysis [145]. Increased FAs levels in circulation can be taken up by skeletal muscle, and the excess of intramuscular FAs was shown to cause several biochemical changes, such as the expression of lipases Atrogin-1 and MuRF67, leading to skeletal muscle atrophy [152,153]. On the other hand, skeletal muscle atrophy may act as positive feedback enhancing AT lipolysis [154]. Thus, the particular crosstalk between AT and muscles in cachexia is an undeniable fact and contributes to the progression of cachexia.

Cachectic adipocytes under some aspects, such as morphologic modifications, decrease in lipid content [25], decrease in expression of C/EBPα [27] and leptin [79], as well as proinflammatory phenotype [65,66,72] and induction of ECM remodeling [27,65,93], resemble CAAs [155] (Table 1 and Table 3). Also, characteristics of cancer cachexia (i.e., decreased glucose metabolism along with decreased lipogenic rate [32]) were observed in CAAs. There are also important features of cachectic adipocytes not seen in CAAs, such as upregulation of lipolytic enzymes (*HSL* or *ATGL*) [35], making them similar to adipocytes in obesity. During AT lipolysis, ATGL and HSL hydrolyze stored triglycerides and produce FFAs and glycerol. In line with this, cachectic—similarly to obese patients—manifests high levels of circulating FFAs, glycerol, and triacylglycerol [156,157,158].

A very reasonable question arises, namely, whether excess body fat and obesity can contribute to cancer cachexia occurrence and worsening patient prognosis. As i) illnesses associated with insulin resistance, such as obesity, exhibit increases in whole-body protein degradation, ii) insulin resistance is associated with muscle fat accumulation, and iii) high-fat diet enhances muscle protein catabolism associated with an increase in plasma FAs and a decrease in plasma adiponectin, the implication of obesity in cancer cachexia is an entirely plausible scenario [159]. Body composition, defined as the proportions and the distribution of lean and fat tissues, is an emergent issue in clinical oncology. Occurrence of sarcopenia, a severe muscle depletion, is most easily missed in obese patients. Coexistence of obesity and sarcopenia, termed “sarcopenic obesity”, are highly prevalent [160]. Mechanisms of muscle loss observed in patients with sarcopenic obesity may involve AT homeostasis and thus likely overlap with mechanisms of cancer cachexia. Obesity was shown to be associated with a decrease in circulating adiponectin and increased IL-6 and FFAs levels (Table 3). These futures were shown to trigger muscle protein loss with concomitant accumulation of fat in muscle fibers [159].

Tumor cells are highly energy-demanding, thus energy and metabolic intermediates such as FFAs released from AT in both obese and cachectic are desired substrates required to sustain their proliferation. However, certain cancers, along with high rate lipolysis, were shown to induce white-to-brown transdifferentiation, named “browning’’, mediated by UCP1, resulting in enhanced fat energy burning with concomitant fat tissue wasting [31]. Paradoxically, this means that energy that could be consumed by tumor cells is burned by thermogenic reactions in adipocytes. In the murine prostate cancer model, p62 emerged as the main regulator of adipocyte metabolic health (supporting browning and adipogenesis). p62 was shown to guard the fat–tumor interaction, as its loss in adipocytes severely inhibited adipocyte browning, allowing establishment of a symbiotic interaction based on adipocyte low energy utilization in favor of tumor needs. In fact, also in BC, p62 levels were reduced in the stroma of several tumors, and its loss resulted in increased tumorigenesis [161].

The process of cancer induced adipocyte browning is largely described in cancer cachexia and concerns adipocytes in the whole body, but this phenomenon has been also observed in the tumor microenvironment. Indeed, higher browning of adipocytes was noted in AT adjacent to BCs when compared to benign lesions [30]. Highly UCP1 expressing cancer-associated fibroblasts were shown to have tumor promoting effects via the generation of high-energy mitochondrial fuels (such as ketone bodies). In fact, this means that even activation of the stroma towards energy consumption (browning) may have a pro-tumorigenic effect through the production of alternative and efficient energy substrates. In general, both adipocytes activation in the tumor vicinity as well as adipocytes extreme modifications observed in cancer cachexia, together with their similarities and differences, are certainly not negligible complications of tumor progression, as cancer cells induce and exploit local and systemic functions for its purpose.

While it seems possible that restoring AT homeostasis by targeting these areas of dysfunction would contribute to further improvements in patient-centered outcomes, current clinical trials investigating potential therapeutic agents for patients with cancer associated cachexia do not directly address the contributions of white, beige, and brown AT to cancer cachexia [154].

## 7. Conclusions

Adipocytes are active players in the development and the progression of BCs. Through the secretion of several stimuli, they create a permissive microenvironment for tumor growth. In the current review, we compared adipocytes involved in three pathologies—obesity, BC, and cachexia. In our opinion, such a comparison may help to understand the way adipocytes support tumor growth and, above all, it can hopefully bring us closer to understanding how cancer cells introduce modifications in AT not found in normal AT. Adipocyte characteristics in obesity are similar to those detected in CAAs of invasive tumors, raising the possibility that adipocytes support tumor development and progression in obese people due to their already altered phenotype. At first glance, such different adipocytes observed in obesity and cachexia turned out to be very similar, especially in terms of metabolic disturbances and pro-inflammatory features, hence becoming dangerous partners of tumor progression. Importantly, altered phenotypes of CAAs and cachectic adipocytes are the results of tumor direct/indirect actions, local and systemic, respectively. Indeed, they both have detrimental effects when it comes to tumor progression and patient prognosis.

Adipocytes, as active player in tumor progression, are emerging as a new target in treating BC. New drugs can be developed to target adipocytes and/or cancer cells to block the adipocyte–tumor cells’ vicious cycle from inducing cancer progression. In this context, further research is required to understand the mechanism(s) by which tumor cells modify adipocytes. Tumor-derived signals to adipocytes might be those used by tumor cells to continuously communicate with and activate stromal cells in the bone marrow or in distant organs in order to progress into metastasis. Moreover, blocking such signals could help in countering tumor-derived adipocyte dysfunction such as cachexia. On the other hand, understanding the efficacy of adipocyte-derived factor inhibition on cancer progression could represent a new strategy to treat cancer, especially in obese patients. Curiously, adipocytes have been also exploited to be the final result of a trans-differentiation process induced in BC cells to reduce BC progression [162]. Indeed, it was recently demonstrated that cancer cells undergoing an EMT program to adapt to changing signals from the microenvironment and to escape drug treatments acquired cellular plasticity that could be exploited to force trans-differentiation of BC cells into bona fide postmitotic adipocytes. This treatment was demonstrated to inhibit cancer metastasis in several preclinical models. Based on the relevance of the interaction between tumor cells and adipocytes in BC progression reviewed above, a potential contribution of the cancer cell-derived adipocytes to tumor progression warrants further investigation before the use of this strategy as a therapeutic approach.

## Figures and Tables

**Table 1:** Comparison of adipocyte features in obesity, cachexia and after interaction with tumor cells.

Characteristic	Obesity	CAAs		Cachexia
		In Vivo	In Vitro	
**Size/TG stores**	↑ A size [23]	↓ A size [19] and lipid droplet size [24]	↓ lipid droplets size and number [19,24]	↓ A size and TG content [25]
**Adipogenesis regulators**	↓ C/EBPα and PPARγ [26]	-	↓ PPARγ and C/EBPα [19,24]	↓ PPARγ and C/EBPα [25,27]
**UCP1** **(browning)**	↑ in brown AT [28] ↓ in white AT [29]	↑ [30]	-	↑ [31]
**Glucose transport**	↓ *GLUT4* [28]	-	↓ *GLUT4* and *IRS1* [24]	↓ *GLUT4* [32]
**Lipogenesis**	↑ in white AT [33]	↓ (↓*HOXC8*, *HOXC9*, *FABP4*, and *HSL*) [30]	↓ (↓ *FABP4*, *HSL*, *ATGL*, *CIDEA,* and *FASN*) [24]	↓ (↓ SREBP-1c) [32]
**Lipolytic activity (*ATGL*/*HSL*)**	↑ in subc. A [34]	↓ *HSL* [30]	↓ *HSL* [19]	↑ *HSL* [35]

A: adipocytes; AT: adipose tissue; CAAs: cancer associated adipocytes; subc: subcutaneous; TG: triglycerides.

**Table 2:** Pro-tumorigenic effect of the adipocyte-tumor cell crosstalk.

Feature	Effect	Adipocyte Comments	Ref
**Proliferation**	↑ in ER+ (MCF-7, ZR75-1, T47-D) cells by mature A = in ER+ cells by preadipocytes = in ER- (MMT 060562) cells by mature A	Primary mature subcutaneous rat A and preadipocytes 7 days of direct contact in 3D collagen gel	[61]
	↑ in SUM159PT by mature A = in ZR 75.1, 67NR, 4T1 by mature A	3T3-F442A cell line 3 days coculture in transwell	[19]
	↑ in BC cell lines (MCF7, MDA-MB-231, N-453, N-435S, -468) by mature A	Mature 3T3-L1 2 days coculture in transwell	[60]
**Tumor growth**	EO771 cells injected in brown AT > white AT > subcutaneous AT	Dorsal subcutaneous fat, inguinal white AT, interscapular brown AT of C57BL/6 mice	[62]
	↑ SKOV3ip1 ovarian cancer cells injected with A vs. tumor cells alone	Human primary omental A Nude mice	[21]
**EMT/** **stemness**	↑ E-CAD expression in T47D and MCF-7 cells by preadipocytes = E-CAD in ZR75-1 and MMT 060562 cells by preadipocytes = E-CAD expression in T47D, MCF-7, ZR75-1 and MMT 060562 cells by mature A	Primary mature subcutaneous rat A and preadipocytes 7 days of direct contact in 3D collagen gel	[61]
	↑ change in morphology in MCF-7, MDA-MB-435S, -231 cells by mature A = in MDA-MB-453, -468 cells ↓ E-CAD in MCF7 cells by mature A ↑ Vim in MCF-7, MDA-MB-231 and-435S cells by mature A	3T3-L1 A 2 days coculture in transwell	[60]
**Migration/** **Invasion**	↑ invasion of ZR 75.1, 67NR, SUM159PT, 4T1 cells by mature A ↑ in BC cell lines by CM from CAAs = in BC cell lines by CM from “naive” A ↑ number of metastases in BALB/c mice injected with 4T1 cells previously cultivated with A	3T3-F442A cell line Indirect coculture/CM	[19]
	↑ MCF7, MDA-MB-231, -468, -453, -435S) by mature A ↑ TN (MDA-MB-231, -468, -435S) > ER+ MCF7, HER2+ MDA-MB-453	3T3-L1 A 2 days coculture in transwell	[60]
**Metabolic reprogramming**	↑ accumulation of lipids in T47D and MDA-MB-231 in coculture with A	Human primary omental A	[21]

A: adipocytes; AT: adipose tissue; CAA: Cancer associated adipocytes; CM: conditioned medium; EMT: epithelial mesenchymal transition; ER: estrogen receptor.

**Table 3:** Molecules at the basis of the pro-tumorigenic effect of adipocytes in obesity, cachexia, and after interaction with BC.

Molecules	Obesity	CAAs		Cachexia
		In Vivo	In Vitro	
**TNF-α**	↑ [64]	↑ [30]	↑ [19]	↑ [65,66]
**IL-6**	↑ [67,68]	↑ [19]	↑ [19]	↑ [65,66]
**IL-1β**	↑ [69]	-	↑ [19]	↑ [66]
**CCL2**	↑ [70]	-	↑ [71]	↑ [66,72]
**CCL5**	↑ [73]	↑ [74]	-	-
**Aromatase**	↑ [75]	↑ [76]	-	-
**Leptin**	↑ [77,78]	↑ [30]	↓ [24]	↓ [79] *
**Adiponectin**	↓ [80] *	↓ [19,30]	↓ [19,24]	↑ [81] *
**Resistin**	↑ [67] *	↑ [20]	↓ [19,24]	-
**Autotaxin**	↑ [82,83,84]	↑ [85]	-	-
**HGF**	↑ [86]	↑ [20]	-	-
**IGF-1**	↑ [87,88]	-	-	-
**PAI-1**	↑ [89,90]	↑ [20]	↑ [19]	-
**COL6A3**	↑ [91]	↑ [20]	-	-
**MMP11**	-	↑ [20]	↑ [19,24]	-
**MMPs**	↑ [92]	↑ [20]	↑ (MMP9, [24])	↑ (MMP2 and MMP9, [93])
**Collagen**	↑ (fibrosis, [94])	-	↑ (COL I, [37])	↑ (COL I, III, VI, [27,65])

* Data derived from analyses in plasma/serum and not in AT or adipocytes.
